# Unveiling the dual nature of late-onset systemic lupus erythematosus: A cross-sectional study

**DOI:** 10.1515/rir-2025-0028

**Published:** 2025-12-27

**Authors:** Fengyun Lu, Dongyu Li, Xiaoxuan Sun, Qiang Wang, Chun Ouyang

**Affiliations:** Department of Rheumatology, The First Affiliated Hospital with Nanjing Medical University, Nanjing 210029, Jiangsu Province, China; Department of Nephrology, The First Affiliated Hospital with Nanjing Medical University, Nanjing 210029, Jiangsu Province, China

**Keywords:** systemic lupus erythematosus, late-onset, clinical manifestations, two-step clustering

## Abstract

**Background and Objective:**

Systemic lupus erythematosus (SLE) is a complex, heterogeneous autoimmune disease whose presentation can vary widely with patient age. While SLE in young adults has been extensively characterized, less is known about phenotypes of late-onset SLE.

**Methods:**

This study aimed to characterize the features of late-onset SLE patients in a Chinese cross-sectional study. Patients diagnosed with SLE at age 50 years or older were classified as having late-onset SLE. Early-onset SLE patients from the same cohort were included as controls. Demographic, clinical, and laboratory data were collected, and a two-step cluster analysis was employed to categorize late-onset SLE patients.

**Results:**

A total of 141 patients (27.48%) were classified as late-onset SLE. The onset of lupus in late-onset patients is more insidious, they exhibited lower systemic lupus erythematosus disease activity index-2000 (SLEDAI-2K) scores, and had significantly fewer instances of fever, mucocutaneous, and positive of antibodies compared to early-onset SLE (all *P* values < 0.05). However, late-onset SLE patients had a higher prevalence of comorbidities, particularly Sjögren’s syndrome (*P* < 0.001). Additionally, late-onset SLE was associated with a high frequency of interstitial lung disease (ILD) and thrombotic events (*P <* 0.001, *P <* 0.001; respectively). Two distinct clusters of late-onset SLE patients were identified. Cluster 1 was characterized by the presence of SLE-specific autoantibodies such as anti-double stranded DNA (anti-dsDNA), anti-Smith (anti-Sm) with higher SLEDAI-2K scores (11.8 ± 5.2). In contrast, cluster 2 presented with a high frequency of anti-Sjögren syndrome antigen A (anti-SSA) antibodies and Sjögren’s syndrome with a significantly lower SLEDAI-2K scores (8.8 ± 5.4) at diagnosis.

**Conclusion:**

This study refines our understanding of late-onset SLE by delineating two subgroups and suggests that age-stratified approaches to diagnosis and management may improve patient care.

## Introduction

Systemic lupus erythematosus (SLE) is an autoimmune disease that can present at any age, with diverse clinical manifestations. Although SLE most commonly begins in young women, a significant minority of cases occur later in life: studies estimate that roughly 10%–25% of SLE patients have disease onset at age ≥ 50 years.^[1]^ In the literature, “late-onset” SLE is frequently defined by a cutoff of 50 years- an age threshold that roughly corresponds to the postmenopausal period- even though some authors have proposed higher cut-offs (such as 60 years).

Clinically, late-onset SLE is known to differ from younger-onset disease in several respects. Older patients tend to present more insidiously, with nonspecific symptoms (such as fatigue, weight loss, arthralgias) rather than the classic malar rash or severe nephritis seen in many younger patients.^[2,3]^ In addition, comorbid conditions accumulate with age: previous reports have noted that older SLE patients have higher rates of hypertension, cardiovascular disease, diabetes, and other autoimmune illnesses compared to younger patients.^[4]^ However, the interplay between SLE manifestations and age-related comorbidities in late-onset SLE is not fully understood. In particular, it is unclear whether distinct subgroups (phenotypic clusters) exist among older-onset patients.

To address this, we conducted a cross-sectional study of patients diagnosed with SLE according to standard criteria, comparing those with disease onset at age ≥ 50 (late-onset SLE) to a reference group with onset at ages 18–49 years (adult-onset SLE). Our goal was to clarify the “dual nature” of late-onset SLE and elucidate how these subgroups might influence clinical practice.

## Methods

### Patients

We conducted a cross-sectional study involving 513 newly diagnosed SLE patients between October 2019 and July 2025 at The First Affiliated Hospital with Nanjing Medical University. Patients were included based on either the 1997 revised American College of Rheumatology classification criteria ^[5]^ or the 2012 Systemic Lupus International Collaborating Clinics criteria ^[6]^ for SLE. To investigate the risk factors and clinical characteristics associated with late-onset SLE, patients were stratified into two groups: early-onset SLE (diagnosis age between 18 and 49 years) and late-onset SLE (diagnosis age ≥ 50 years). The study was approved by the institutional ethics committee, and all patients provided informed consent (2024-SR-221).

### Data Collection

At the time of SLE diagnosis, data were collected on a range of variables, including demographic, clinical and laboratory manifestations, comorbidities, treatments (hydroxychloroquine, daily prednisone intake, and immunosuppressive agents).

Laboratory data within 10 days before or at the time of hospitalization were recorded, including complete blood cell count, 24-hour proteinuria, estimated glomerular filtration rate (eGFR), complement 3 (C3) and complement 4 (C4), C-reactive protein (CRP), anti-nuclear antibodies (ANA) by Indirect immunofluorescence assay (IFA), anti-phospholipid antibodies (including anti-cardiolipin and anti-β2-glycoprotein-1[anti-β2GPI]; by enzyme-linked immunosorbent assay [ELISA]) and anti-double stranded DNA (anti-dsDNA) (by ELISA or IFA). Additionally, autoantibodies such as anti-Sm, anti-small nuclear ribonucleoprotein (anti-snRNP), anti-nucleosome, anti-histone, anti-Sjögren syndrome antigen A or Ro60 (anti-SSA/Ro60), anti-Sjögren syndrome antigen A or Ro52 (anti-SSA/Ro52), anti-Sjögren syndrome antigen B or La (anti-SSB/La) were assessed using immunoblotting techniques. Interstitial lung disease (ILD) was evaluated using chest high-resolution computed tomography (HRCT) scans.^[7]^ A confirmed diagnosis of pulmonary hypertension (PAH) *via* Right Heart Catheterization (RHC), the diagnostic criteria for PAH were as follows: mean pulmonary arterial pressure (mPAP) ≥ 20 mmHg as measured by hemodynamic (RHC), pulmonary arterial wedge pressure (PAWP) ≤ 15 mmHg, and pulmonary vascular resistance (PVR) ≥ 2 Wood units.^[8]^ Disease activity was assessed using two standardized measures: (1) SLE Disease Activity Index 2000 (SLEDAI-2K) score;^[9] (^2) patient global assessment (PGA).^[10]^ All variables were analyzed as observed, with no imputation of missing data.

### Statistical Analysis

Statistical analysis and data visualization were performed using R software (version 4.4.2; R Foundation for Statistical Computing, Vienna, Austria) and GraphPad Prism software (version 10.1.2; GraphPad Software, San Diego, CA, USA). Continuous variables with normal distribution are presented as mean ± standard deviation, while those with non-normal distribution are presented by median (Q1, Q3). The *t* test or Mann-Whitney *U* test was employed for continuous variables, while Chi-square test or Fisher’s exact test was used for categorical variables. Multivariate logistic regression analyses were performed to control for age as a potential confounder in the comparison of comorbidities between early- and late-onset SLE groups. *P <* 0.05 was considered statistically significant.

To categorize subtypes of late-onset SLE patients, a two-step cluster analysis was performed using IBM SPSS Statistics software (version 24.0; IBM Corporation, Armonk, NY, USA).^[11]^ In the first phase, variables are grouped into “pre-clusters”, which are then clustered using hierarchical clustering methods. During the pre-clustering phase, the two-step algorithm applies Euclidean distance to continuous variables and log-likelihood distance for discrete variables. In our analysis, the log-likelihood distance measure was used due to the binary nature of the variables. In the second phase, clusters are formed by applying hierarchical clustering based on the log-likelihood distance measure. The optimal number of clusters determined by the Bayesian information criterion (BIC) and the quality of clustering was evaluated using the silhouette score, which ranges from-1 to +1. Higher silhouette scores indicate better clustering quality, with values closer to +1 representing well-separated clusters, while values closer to-1 suggest poor clustering.^[12]^

## Results

**Figure 1:**
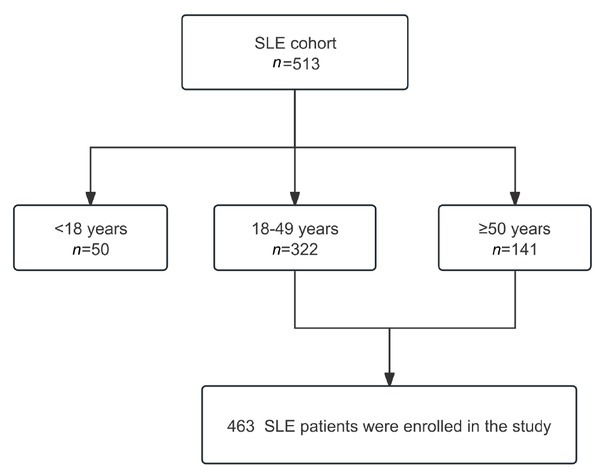
Flow diagram of SLE patient enrollment.

For the present study, we included a total of 513 cases of SLE, comprising 141 late-onset lupus patients and 322 early-onset lupus patients, as shown in Figure 1.

### The Clinical Manifestation of Late-onset and Early-onset SLE Patients

Significant differences were observed between younger and older SLE patients. The female-to-male ratio was 3.4: 1 in the late-onset group and 6.5: 1 in the early-onset group (*P* = 0.012). Disease onset was defined as the interval from the first symptom or sign of lupus to the date the patients diagnosed with SLE fulfilled the classification criteria. There was a significant delay in the diagnosis of SLE among older patients [median 1.5 months (interquartile range [IQR] 1.0–3.1) *vs*. 1.0 months (IQR 0.5–2.1), *P =* 0.002]. Late-onset SLE exhibited significantly lower score of SLEDAI-2K (9.6 ± 5.3 *vs*. 12.3 ± 6.0, *P* < 0.001) and PGA (1.3 ± 0.6 *vs*. 1.5 ± 0.6, *P* < 0.001). Older SLE patients exhibited significantly lower prevalences of anti-Sm (41.84% *vs*. 57.14%), anti-dsDNA (42.55% *vs*. 64.91%), anti-nucleosome (31.91% *vs*. 55.59%), and anti-histone (31.91% *vs*. 48.76%) (all *P <* 0.05). Conversely, they had a significantly higher prevalence of anti-ribosomal P protein (anti-RPP) (16.31% *vs*. 4.35%, *P* < 0.001). The late-onset patients also displayed significantly higher mean complement levels (C3: 0.51 *vs*. 0.42 g/L; C4: 0.11 *vs*. 0.08 g/L, both *P <* 0.001), and lower eGFR (99.66 mL/min/1.73 m^2^ *vs*. 121.26 mL/min/1.73 m^2^, *P <* 0.001).

Late-onset SLE patients demonstrated a distinct clinical profile characterized by significant differences in organ involvement, comorbidities, and treatment patterns. They exhibited a substantially higher prevalence of interstitial lung disease (ILD) (9.22% *vs*. 2.17%, *P* < 0.001) but lower rates of fever (5.67% *vs*. 14.60%, *P* = 0.006) and mucocutaneous involvement (27.66% *vs*. 44.10%, *P* < 0.001). Older patients were significantly more likely to have overlapping connective tissue diseases, particularly Sjögren’s syndrome (26.95% *vs*. 11.18%, *P <* 0.001), systemic sclerosis (4.96% *vs*. 0.93%, *P* = 0.016), and rheumatoid arthritis (5.67% *vs*. 0.93%, *P=* 0.006). Comorbid conditions were markedly more frequent in the older group, including hypertension (30.50% *vs*. 11.80%, *P* < 0.001), diabetes (9.22% *vs*. 0.62%, *P* < 0.001), coronary heart disease (4.26% *vs*. 0.31%, *P* = 0.005). Treatment analysis revealed older patients received hydroxychloroquine less frequently (76.60% *vs*. 90.31%, *P* < 0.001) and biological agents less often (12.77% *vs*. 21.74%, *P* = 0.024), while their prednisone dosing distribution differed significantly (*P* = 0.005), with higher proportions receiving ≥ 1 mg/kg/d (72.34% *vs*. 65.84%). Table 1 provide detailed descriptive characteristics of early-onset and late-onset patients.

**Table 1:** Clinical variables of early-onset and late-onset SLE patients

	18–50 years, *n* = 322	> 50 years, *n* = 141	*P* value
Demographic data			
Female, *n* (%)	279 (86.65)	109 (77.30)	0.012^*^
Age, year, mean ± SD	31.99 ± 9.01	59.13 ± 7.45	< 0.001^*^
Clinical parameters			
Disease onset, month, M (Q1, Q3)	1.0 (0.5, 2.1)	1.5 (1.0, 3.1)	0.002^*^
SLEDAI-2K, mean ± SD	12.3 ± 6.0	9.6 ± 5.3	< 0.001^*^
PGA, mean ± SD	1.5 ± 0.6	1.3 ± 0.6	< 0.001^*^
Laboratory and serology			
Anti-Sm, *n* (%)	184 (57.14)	59 (41.84)	0.002^*^
Anti-dsDNA, *n* (%)	209 (64.91)	60 (42.55)	< 0.001^*^
Anti-snRNP, *n* (%)	186 (57.76)	78 (55.32)	0.625
Anti-nucleosome, *n* (%)	179 (55.59)	45 (31.91)	< 0.001^*^
Anti-histone, *n* (%)	157 (48.76)	45 (31.91)	< 0.001^*^
Anti-SSA/Ro60, *n* (%)	221 (68.63)	87 (61.70)	0.146
Anti-SSA/Ro52, *n* (%)	180 (55.90)	69 (48.94)	0.167
Anti-SSB/La, *n* (%)	123 (38.20)	44 (31.21)	0.149
Anti-RPP, *n* (%)	14 (4.35)	23 (16.31)	< 0.001^*^
Anti-β2GPI moderate to high titers, *n* (%)	30 (11.86)	10 (8.70)	0.366
ACA moderate to high titers, *n* (%)	49 (17.95)	12 (9.02)	0.018^*^
ANA titer			1.000
1:100, *n* (%)	9 (2.80)	4 (2.84)	
≥1:320, *n* (%)	313 (97.20)	137 (97.16)	
C3, g/L, mean ± SD	0.42 ± 0.21	0.51 ± 0.23	< 0.001^*^
C4, g/L, mean ± SD	0.08 ± 0.06	0.11 ± 0.08	< 0.001^*^
PLT, × 10^9^/L, M (Q1, Q3)	152 (104, 219)	123 (77, 195)	0.002^*^
WBC, × 10^9^/L, M (Q1, Q3)	4.08 (2.93, 6.17)	4.33 (3.01, 6.47)	0.559
Hb, g/L, mean± SD	98.76 ± 20.45	97.99 ± 21.25	0.710
CRP, mg/L, M (Q1, Q3)	3.81 (1.71, 9.17)	6.09 (3.10, 14.00)	< 0.001^*^
24-h proteinuria > 0.5 g, *n* (%)	173 (53.73)	67 (47.52)	0.219
eGFR, mL/min/1.73 m^2^, M (Q1, Q3)	121.26 (102.49, 130.62)	99.66 (72.43, 107.47)	< 0.001^*^
CD19+B cells, %, M (Q1, Q3)	19.60 (13.90, 28.00)	17.70 (12.43, 27.05)	0.171
Systemic involvement			
Fever, *n* (%)	47 (14.73)	8 (5.67)	0.006^*^
Mucocutaneous, *n* (%)	142 (44.10)	39 (27.66)	< 0.001^*^
Renal involvement, *n* (%)	175 (54.35)	69 (48.94)	0.283
Vasculitis, *n* (%)	21 (6.52)	4 (2.84)	0.106
Arthralgia, *n* (%)	62 (19.25)	21 (14.89)	0.260
Serosal, *n* (%)	124 (38.51)	55 (39.01)	0.919
CNS, *n* (%)	27 (8.39)	7 (4.96)	0.194
ILD, *n* (%)	7 (2.17)	13 (9.22)	< 0.001^*^
PAH, *n* (%)	28 (8.70)	12 (8.51)	0.948
Myocarditis, *n* (%)	56 (17.39)	22 (15.60)	0.636
Gastrointestinal, *n* (%)	25 (7.76)	6 (4.26)	0.164
Thrombotic events, *n* (%)	14 (4.35)	28 (19.86)	< 0.001^*^
Treatments			
GC (max. dosage), *n* (%)			0.005^*^
Pulse therapy	57 (17.70)	15 (10.64)	
≥1 mg/kg/d	212 (65.84)	102 (72.34)	
0.5–1 mg/kg/d	45 (13.98)	12 (8.51)	
< 0.5 mg/kg/d	7 (2.17)	9 (6.38)	
Unused	1 (0.31)	3 (2.13)	
Immunosuppression, *n* (%)	271 (84.16)	119 (84.40)	0.949
Hydroxychloroquine, *n* (%)	289 (90.31)	108 (76.60)	< 0.001^*^
Biologicals, *n* (%)	70 (21.74)	18 (12.77)	0.024^*^
Combined other CTD			
Sjögren’s syndrome, *n* (%)	36 (11.18)	38 (26.95)	< 0.001^*^
Antiphospholipid syndrome, *n* (%)	28 (8.70)	11 (7.80)	0.750
Systemic sclerosis, *n* (%)	3 (0.93)	7 (4.96)	0.016^*^
Rheumatoid arthritis, *n* (%)	3 (0.93)	8 (5.67)	0.006^*^
ANCA associated vasculitis, *n* (%)	2 (0.62)	4 (2.84)	0.135
Comorbid conditions			
Hypertension, *n* (%)	38 (11.80)	43 (30.50)	< 0.001^*^
Diabetes, *n* (%)	2 (0.62)	13 (9.22)	< 0.001^*^
Coronary heart disease, *n* (%)	1 (0.31)	6 (4.26)	0.005^*^
Tumor, *n* (%)	6 (1.86)	7 (4.96)	0.120

*, statistically significant; ANA, anti-nuclear antibody; anti-dsDNA, anti-double-stranded DNA; anti-Sm, anti-Smith; anti-snRNP, anti-small nuclear ribonucleoprotein; anti-SSA/Ro60, anti-Sjögren syndrome antigen A or Ro60; anti-SSA/ Ro52, anti-Sjögren syndrome antigen A or Ro52; anti-SSB/La, anti-Sjögren’s syndrome-related antigen B or La; anti-RPP, anti-ribosomal P protein; anti-β2 GPI, anti-β2-glycoprotein I; ACA, anti-cardiolipin antibody; CRP, C-reactive protein; PLT, platelet count; WBC, white blood cell count; Hb, hemoglobin; eGFR, estimated glomerular filtration rate; CNS, central nervous system; ILD, interstitial lung disease; PAH, pulmonary arterial hypertension; GC, glucocorticoids; CTD, connective tissue disease. Notes: Anti-β2 GPI moderate/high titers defined as >40 U/mL. ACA moderate/high titers defined as >40 GPL/MPL (GPL: IgG phospholipid units; MPL: IgM phospholipid units). Renal involvement was defined as 24-hour proteinuria >0.5 g and/or positive renal biopsy. Myocarditis was diagnosed by cardiac magnetic resonance and/or electrocardiogram and/or serum cardiac markers.

### Two Distinct Clusters of Late-onset SLE Patients

Our analysis revealed a distinct autoantibody profile in patients with late-onset SLE compared to those with early-onset. Multiple studies demonstrate that distinct autoantibody clusters associate with heterogeneous clinical phenotypes in SLE. To further delineate the phenotypic diversity among late-onset SLE patients, we performed two-step cluster analysis on 12 autoantibodies: ANA, anti-dsDNA, anti-nucleosome, anti-histone, anti-Sm, anti-SSA/Ro52, anti-SSA/Ro60, anti-SSB/La, anti-snRNP, anti-RPP, anti-β2 GPI, ACA. This analysis included 114 patients with late-onset SLE; 27 SLE patients were excluded due to missing data for these auto-antibodies. The cluster analysis identified two distinct patient clusters, with the solution demonstrating moderate cohesion (average silhouette measure = 0.4), which is similar or higher than those reported in several other studies,^[11,13,14]^ indicating a reasonable structure for the clusters.

**Figure 2:**
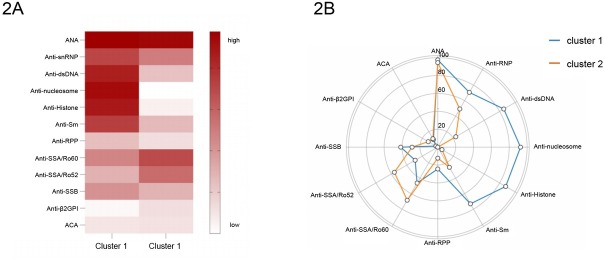
A, Autoantibody clusters of patients with Late-onset SLE in the study. Heatmap shows the frequencies for each autoantibody by cluster. B, Radar plots show the frequencies for each autoantibody by cluster.

The heatmap in Figure 2A illustrates the two distinct clusters, with bright red indicating high frequencies of a given phenotype and white indicating very low incidences. Cluster 1 (*n* = 41) exhibited a classical lupus-specific autoreactivity profile characterized by significantly higher frequencies of anti-dsDNA (85.37% *vs*. 23.29%, *P* < 0.001), anti-Sm (73.17% *vs*. 26.03%, *P* < 0.001), anti-nucleosome (92.68% *vs*. 0.00%, *P* < 0.001), and anti-histone antibodies (87.80% *vs*. 5.48%, *P* < 0.001) compared to Cluster 2 (*n* = 73). Conversely, Cluster 2 demonstrated a phenotype with significantly elevated anti-SSA/Ro60 (68.49% *vs*. 46.34%, *P* = 0.020) and anti-SSA/Ro52 antibodies (56.16% *vs*. 29.27%, *P* = 0.006), Supplementary Table S1, Figure 2B.

Cluster 1 demonstrates significant hypocomplementemia, with markedly lower mean C3 (0.39 *vs*. 0.51 g/L, *P* = 0.002) and C4 (0.07 *vs*. 0.11 g/L, *P* = 0.017) levels compared to Cluster 2. This profound complement consumption signifies more active immune complex-mediated pathogenesis, aligning with their significantly higher systemic disease activity (SLEDAI-2K: 11.8 *vs*. 8.8, *P* = 0.004; PGA: 1.5 *vs*. 1.2, *P* = 0.003). Cluster 2, while exhibiting less complement activation, shows a significantly higher prevalence of pulmonary arterial hypertension (PAH: 12.33% *vs*. 0.00%, *P* = 0.048), ILD (15.07% *vs*. 2.44%, *P* = 0.073) and greater association with Sjögren’s syndrome (34.25% *vs*. 14.63%, *P* = 0.024), suggesting divergent autoantibody specificities or pathogenetic mechanisms (Table 2).

**Table 2:** Baseline parameters and clinical outcomes of patients among autoantibody clusters

	cluster 1 (*n* = 41)	cluster 2 (*n* = 73)	*P* value
Demographic data			
Female, *n* (%)	33 (80.49)	55 (75.34)	0.530
Age, year, mean ± SD	57.90 ± 7.18	59.84 ± 7.82	0.115
Clinical parameters			
Disease onset, month, M (Q1, Q3)	1.13 (0.70, 2.67)	1.47 (0.77, 3.13)	0.871
SLEDAI-2K, mean ± SD	11.8 ± 5.2	8.8 ± 5.4	0.004^*^
PGA, mean ± SD	1.5 ± 0.6	1.2 ± 0.6	0.003^*^
Laboratory and serology			
C3, g/L, M (Q1, Q3)	0.39 (0.21, 0.55)	0.51 (0.38, 0.67)	0.002^*^
C4, g/L, M (Q1, Q3)	0.07 (0.04, 0.12)	0.11 (0.07, 0.15)	0.017^*^
PLT, × 10^9^/L, M (Q1, Q3)	126.0 (78.0, 195.0)	121.0 (75.0, 192.0)	0.682
WBC, × 10^9^/L, M (Q1, Q3)	3.72 (2.84, 5.21)	5.14 (3.19, 7.17)	0.016^*^
Hb, g/L, mean± SD	95.88 ± 14.99	99.27 ± 21.74	0.328
CRP, mg/L, M (Q1, Q3)	8.09 (5.32, 14.55)	5.50 (3.09, 13.02)	0.086
24-hour proteinuria > 0.5 g, *n* (%)	21 (51.22)	26 (35.62)	0.104
eGFR, mL/min/1.73 m^2^, M (Q1, Q3)	102.28 (72.52, 107.29)	100.66 (83.65, 107.35)	0.966
Systemic involvement			
Fever, *n* (%)	5 (12.20)	3 (4.11)	0.215
Mucocutaneous, *n* (%)	15 (36.59)	22 (30.14)	0.480
Renal involvement, *n* (%)	20 (48.78)	28 (38.36)	0.279
Vasculitis, *n* (%)	1 (2.44)	2 (2.74)	1.000
Arthralgia, *n* (%)	10 (24.39)	11 (15.07)	0.218
Serosal, *n* (%)	19 (46.34)	29 (39.73)	0.492
CNS, *n* (%)	3 (7.32)	4 (5.48)	1.000
ILD, *n* (%)	1 (2.44)	11 (15.07)	0.073
PAH, *n* (%)	0 (0.00)	9 (12.33)	0.048^*^
Myocarditis, *n* (%)	7 (17.07)	14 (19.18)	0.781
Gastrointestinal, *n* (%)	3 (7.32)	2 (2.74)	0.504
Thrombotic events, *n* (%)	10 (24.39)	16 (21.92)	0.763
Treatments			
GC (max. dosage), *n* (%)			0.065
Pulse therapy	0 (0.00)	1 (1.37)	
≥1 mg/kg/d	4 (9.76)	11 (15.07)	
0.5–1 mg/kg/d	36 (87.80)	48 (65.75)	
< 0.5 mg/kg/d	1 (2.44)	6 (8.22)	
Unused	0 (0.00)	7 (9.59)	
Immunosuppression, *n* (%)	32 (78.05)	65 (89.04)	0.114
Hydroxychloroquine, *n* (%)	35 (85.37)	55 (75.34)	0.208
Biologicals, *n* (%)	7 (17.07)	7 (9.59)	0.243
Combined other CTD			
Sjögren’s syndrome, *n* (%)	6 (14.63)	25 (34.25)	0.024^*^
Antiphospholipid syndrome, *n* (%)	4 (9.76)	7 (9.59)	1.000
Systemic sclerosis, *n* (%)	0 (0.00)	6 (8.22)	0.147
Rheumatoid arthritis, *n* (%)	1 (2.44)	2 (2.74)	1.000
ANCA associated vasculitis, *n* (%)	0 (0.00)	4 (5.48)	0.319
Comorbid conditions			
Hypertension, *n* (%)	7 (17.07)	22 (30.14)	0.124
Diabetes, *n* (%)	5 (12.20)	4 (5.48)	0.361
Coronary heart disease, *n* (%)	1 (2.44)	4 (5.48)	0.776
Tumor, *n* (%)	1 (2.44)	4 (5.48)	0.776

*, statistically significant; SLEDAI-2K, Systemic Lupus Erythematosus Disease Activity Index 2000; PGA, Patient Global Assessment; C3, complement 3; C4, complement 4; PLT, platelet count; WBC, white blood cell count; Hb, hemoglobin; CRP, C-reactive protein; eGFR, estimated glomerular filtration rate; CNS, central nervous system; ILD, interstitial lung disease; PAH, pulmonary arterial hypertension; GC, glucocorticoids; CTD, connective tissue disease.

## Discussion

This study delineates the distinct phenotype of newly diagnosed late-onset SLE compared to early-onset disease. We chose age 50 as the cutoff for late-onset based on its common use in the literature,^[2,4,15, 16, 17, 18]^ which approximates the menopausal transition and the onset of immune aging. We acknowledge that this choice is somewhat arbitrary- indeed, some studies have proposed 60 as an alternative threshold^[19]^ -and that any dichotomous cutoff on a continuous variable has limitations. Studies show SLE varies by age of onset. Early-onset is more severe. Late-onset has more pulmonary/ serositis involvement but less malar rash, photosensitivity, arthritis, and nephritis.^[7,20,21]^ To address whether alternative age thresholds would alter our conclusions, we conducted a subgroup analysis comparing patients aged 50–60 years (*n* = 85) *versus* > 60 years (*n* = 56) within our late-onset SLE cohort. We found that while there were expected differences in age-related comorbidities (*e.g*., coronary heart disease) and slight variations in disease activity scores, the core clinical, serological, and organ-involvement profiles remained largely consistent across the two age subgroups. Notably, autoantibody patterns—central to our cluster analysis—did not differ significantly (Supplementary Table S2). These findings reinforce that using 50 years as the cutoff captures a phenotypically distinct group consistent with the literature, and supports the robustness of our identified clusters.

In alignment with previous reports, our study also confirmed that juvenile-onset SLE tends to present with a more severe phenotype, late-onset SLE demonstrated attenuated disease activity, reduced classic manifestations (such as mucocutaneous involvement), and distinct serological profiles compared to early-onset disease. This attenuated clinical presentation contributed to a significant diagnostic delay (median 1.47 *vs*. 1.03 months, *P* = 0.002). For instance, Cervera *et al*. described reduced renal involvement in late-onset SLE (22% *vs*. 41% in early-onset) and limited pulmonary manifestations (9%) in this subgroup.^[22]^ These findings appear partly consistent with observations from a Korean cohort which reported a relatively low proportion (13.5%) of lupus nephritis (LN) among late-onset patients.^[4]^ In our study found a substantial proportion of late-onset patients presented with LN (69/141, 48.94%). However, this rate was not significantly different from that in the early-onset group within our cohort (54.35%, *P* = 0.283), suggesting that the risk of renal involvement in Chinese SLE patients may persist into older age. The marked discrepancy in late-onset LN prevalence suggests potential ethnic variations and underscores the imperative for vigilant renal monitoring across all age groups in Chinese SLE patients.

Consistent with previous reports,^[2,23,24]^ our study confirms a higher burden of comorbidities in patients with late-onset SLE. After multivariate adjustment for age, only diabetes mellitus remained independently associated with late-onset SLE (*P* = 0.005). However, the notably wide confidence interval suggests limited. After multivariate adjustment for age, only diabetes mellitus remained independently associated with late-onset SLE (*P* = 0.005) precision in this estimate, likely due to the small number of events, and thus this finding should be interpreted with caution. In contrast, the increased prevalence of hypertension appeared to be primarily driven by advancing age itself rather than by late-onset SLE status. Although numerically more cases of coronary heart disease were observed in the late-onset group, the low event count precluded stable multivariate modeling, Supplementary Table S3. These observations highlight the need for further validation in larger prospective cohorts to better delineate the independent contributions of age and SLE phenotype to comorbidity profiles. Notably, ILD occurred significantly more frequently (9.22% *vs*. 2.17%, *P <* 0.001), potentially related to advancing age, immunosenescence, and an association with SLE-Sjögren’s overlap syndrome conferring higher ILD risk in this subgroup.^[18]^ These findings highlight the more complex comorbidity burden, distinct organ manifestation patterns (particularly increased ILD), and different therapeutic approaches in late-onset SLE.

Critically, this study demonstrates that late-onset SLE encompasses at least two distinct phenotypic clusters—revealed through our cluster analysis as divergent subgroups masked within the aggregate profile—a novel finding underscoring the disease’s heterogeneity in older adults. Our “lupus-dominant” cluster (Cluster 1) essentially mirrors younger-onset SLE in its clinical and serologic intensity, while our “comorbidity-dominant” cluster (Cluster 2) suggests a syndrome in which age-related health conditions are the prevailing feature and classical SLE manifestations are attenuated. This dual nature is not entirely unexpected: older SLE patients are known to present more subtly, and it is well established that comorbidities accumulate with age. However, by formally identifying clusters, we extend prior knowledge by showing that there is a subgroup of late-onset patients for whom comorbid conditions effectively eclipse lupus symptoms.

Cluster 1 was characterized by specific autoantibodies (anti-dsDNA, anti-Sm) and hypocomplementemia, reflecting a pathogenesis driven by immune complex (IC) deposition and type I interferon (IFN) pathway activation.^[25,26]^ These autoantibodies—highly specific for SLE—form pathogenic ICs that deposit in tissues such as the kidney, activate complement (accounting for low C3/C4), and exacerbate inflammation, leading to severe organ manifestations.^[27]^ This mechanism, which relies on adaptive immunity involving T and B cell collaboration,^[28]^ indicates that a strong lupus-specific autoimmune drive persists in this late-onset subgroup, overriding age-related immunosenescence. Cluster 2 was characterized by a higher prevalence of anti-SSA/Ro antibodies, Sjögren’s syndrome, ILD, and PAH, alongside relatively mild lupus activity. The anti-Ro52 antibody—which is frequently associated with rapidly progressive ILD and poor prognosis in autoimmune diseases such as idiopathic inflammatory myopathy-associated interstitial lung disease (IIM-ILD)—may reflect a shared immunopathogenic mechanism.^[29]^ Mechanistically, anti-Ro52 is closely linked to robust type I interferon activation, promoting innate immune-mediated damage in lung and vascular tissues, thereby contributing to the prominent cardiopulmonary manifestations observed in this cluster. This phenotype is further supported by findings from the Chinese SLE Treatment and Research Group (CSTAR) registry, in which both anti-SSA positivity and older age at onset independently predicted organ damage accumulation in SLE patients.^[30]^ These results suggest that Cluster 2 represents a high-risk subgroup within late-onset SLE, necessitating vigilant monitoring for progressive organ damage—particularly cardiopulmonary complications.

Recognizing this distinction is clinically critical, as it directly informs therapeutic strategy. Patients in the lupus-dominant cluster likely require more aggressive, lupus-specific therapy tailored to particular organ involvement. In contrast, patients in the comorbidity-dominant cluster may benefit from a conservative approach to immunosuppression, coupled with proactive management of age-related comorbidities such as cardiovascular disease and geriatric syndromes. Although these comorbidities are often not SLE-specific, their high prevalence in late-onset patients necessitates careful management to avoid overtreatment. Importantly, older patients frequently exhibit reduced tolerance to immunosuppressive agents; our clustering helps contextualize this by identifying which patients may still require standard lupus regimens versus which patients warrant individualized, lower-intensity therapy. The overarching goal remains to balance treatment—avoiding both inadequate control of lupus activity and excessive intervention that may worsen comorbidities.

Limitations of this study include its retrospective design and dependence on available clinical data. Unmeasured confounders could influence cluster formation. Additionally, our cohort size (and that of some clusters) was modest, which highlighs the challenge of recruiting large late-onset SLE cohorts due to diagnostic delays and heterogeneity. While our clustering approach achieved moderate separation (silhouette score = 0.4), smaller sample sizes may limit the stability of subgroup identification. Nonetheless, the consistency of our clusters with known SLE subgroups, and the novelty of applying cluster analysis to a late-onset cohort, lend credibility to the findings.

## Conclusions

In summary, our analysis of a late-onset SLE study reveals a “dual nature” of the disease in older adults. One subgroup of patients has a clinical profile much like younger-onset lupus, with prominent SLE-specific manifestations, while another subgroup’s profile is dominated by age-related comorbidities and relatively mild lupus activity. This phenotypic heterogeneity has important implications: it suggests that late-onset SLE should not be treated as a single entity, and that clinical management should be personalized according to the patient’s dominant phenotype. By delineating these clusters, we clarify the scientific understanding of SLE across the lifespan and emphasize the need for age-tailored approaches in both research and care.

## Supplementary Material

Supplementary Material Details
