# Supplementary material for: Unveiling the dual nature of late-onset systemic lupus erythematosus: A cross-sectional study
Source: Rheumatol Immunol Res. 2025 Dec 27;6(4):232–9. doi: 10.1515/rir-2025-0028 (PMC12743268; doi:10.1515/rir-2025-0028)
Supplement: Supplementary file 1 — Supplementary Material Details [file rir-2025-0028_sm.pdf]

Supplementary materials

Supplementary Table S1 Comparison of autoantibody frequencies in patients with late-onset SLE by clusters.

**Variables**	**Cluster 1 (*n* = 41)**	**Cluster 2 (*n* = 73)**	***P* value**
Anti-dsDNA, *n* (%)	35 (85.37)	17 (23.29)	<0.001*
Anti-Sm, *n* (%)	30 (73.17)	19 (26.03)	<0.001*
Anti-snRNP, *n* (%)	29 (70.73)	36 (49.32)	0.027*
Anti-nucleosome, *n* (%)	38 (92.68)	0 (0.00)	<0.001*
Anti-histone, *n* (%)	36 (87.80)	4 (5.48)	<0.001*
Anti-RPP, *n* (%)	10 (24.39)	9 (12.33)	0.097
Anti-SSA/Ro60, *n* (%)	19 (46.34)	50 (68.49)	0.020*
Anti-SSA/Ro52, *n* (%)	12 (29.27)	41 (56.16)	0.006*
Anti-SSB/La, *n* (%)	17 (41.46)	21 (28.77)	0.168
Anti-β2 GPI moderate to high titers, *n* (%)	1 (2.44)	9 (12.33)	0.148
ACA moderate to high titers, *n* (%)	4 (9.76)	8 (10.96)	1.000
ANA titer			0.776
1:100, *n* (%)	40 (97.56)	69 (94.52)	
≥1:320, *n* (%)	1 (2.44)	4 (5.48)	

Supplementary Table S2 Comparison of Clinical Characteristics Between SLE Patients Aged 50–60 Years and Those Over 60 Years

**Variables**	**50–60 years (*n* = 85)**	**>60 years (*n* = 56)**	***P* value**
**Demographic data**			
Female, *n* (%)	71 (83.53)	38 (67.86)	0.030*
**Clinical parameters**			
Disease onset, month, M (Q1, Q3)	1.27 (0.67, 2.43)	2.02 (0.88, 3.90)	0.187
SLEDAI-2K, mean ± SD	10.52 ± 5.72	8.16 ± 4.30	0.006*
PGA, mean ± SD	1.36 ± 0.60	1.11 ± 0.45	0.005*
**Laboratory and serology**			
Anti-Sm, *n* (%)	38 (44.71)	21 (37.50)	0.396
Anti-dsDNA, *n* (%)	40 (47.06)	20 (35.71)	0.182
Anti-snRNP, *n* (%)	52 (61.18)	26 (46.43)	0.085
Anti-nucleosome, *n* (%)	27 (31.76)	18 (32.14)	0.962
Anti-histone, *n* (%)	30 (35.29)	15 (26.79)	0.289
Anti-SSA/Ro60, *n* (%)	50 (58.82)	37 (66.07)	0.386
Anti-SSA/Ro52, *n* (%)	38 (44.71)	31 (55.36)	0.216
Anti-SSB/La, *n* (%)	22 (25.88)	22 (39.29)	0.093
Anti-RPP, *n* (%)	13 (15.29)	10 (17.86)	0.687
Anti-β2 GPI moderate to high titers, *n* (%)	9 (13.04)	1 (1.79)	0.048*
ACA moderate to high titers, *n* (%)	11 (13.75)	1 (1.79)	0.035*
ANA titer			0.651

1:100, *n* (%)	81 (95.29)	55 (98.21)	
≥1:320, *n* (%)	4 (4.71)	1 (1.79)	
C3, g/L, mean ± SD	0.49 ± 0.24	0.53 ± 0.22	0.415
C4, g/L, mean ± SD	0.10 ± 0.07	0.12 ± 0.08	0.080
PLT, $\times 10^9$/L, M (Q1, Q3)	125.00 (76.00, 195.00)	119.50 (78.75, 196.25)	0.792
WBC, $\times 10^9$/L, mean ± SD	5.35 ± 4.07	5.61 ± 3.58	0.695
Hb, g/L, mean ± SD	98.71 ± 21.45	96.89 ± 21.08	0.622
CRP, mg/L, M (Q1, Q3)	5.33 (2.87, 12.25)	8.62 (4.69, 15.20)	0.072
24-hour proteinuria>0.5g, *n* (%)	42 (49.41)	25 (44.64)	0.579
eGFR, mL/minute/1.73 m^2, mean ± SD	96.08 ± 22.63	78.55 ± 28.22	<0.001*
$CD19^+$B cells, %, M (Q1, Q3)	16.98 (12.05, 26.83)	20.40 (12.55, 27.55)	0.769
**Systemic involvement**			
Fever, *n* (%)	3 (3.53)	5 (8.93)	0.325
Mucocutaneous, *n* (%)	25 (29.41)	14 (25.00)	0.567
Renal involvement, *n* (%)	35 (41.18)	20 (35.71)	0.889
Vasculitis, *n* (%)	3 (3.53)	1 (1.79)	0.927
Arthralgia, *n* (%)	15 (17.65)	6 (10.71)	0.258
Serosal, *n* (%)	35 (41.18)	20 (35.71)	0.515
CNS, *n* (%)	7 (8.24)	0 (0.00)	0.071
ILD, *n* (%)	8 (9.41)	5 (8.93)	0.923
PAH, *n* (%)	10 (11.76)	2 (3.57)	0.162
Myocarditis, *n* (%)	15 (17.65)	7 (12.50)	0.410
Gastrointestinal, *n* (%)	4 (4.71)	2 (3.57)	1.000
Thrombotic events, *n* (%)	19 (22.35)	9 (16.07)	0.360
**Treatments**			
GC (max. dosage), *n* (%)			0.128
Pulse therapy	12 (14.12)	3 (5.36)	
≥1 mg/kg/day	60 (70.59)	42 (75.00)	
0.5-1 mg/kg/day	8 (9.41)	4 (7.14)	
<0.5 mg/kg/day	5 (5.88)	4 (7.14)	
Unused	0 (0.00)	3 (5.36)	
Immunosuppression, *n* (%)	75 (88.24)	44 (78.57)	0.122
Hydroxychloroquine, *n* (%)	72 (84.71)	36 (64.29)	0.005*
Biologicals, *n* (%)	10 (11.76)	8 (14.29)	0.661
**Combined other CTD**			
Sjögren’s syndrome, *n* (%)	18 (21.18)	20 (35.71)	0.057
antiphospholipid syndrome, *n* (%)	11 (12.94)	0 (0.00)	0.013*
systemic sclerosis, *n* (%)	5 (5.88)	2 (3.57)	0.824
rheumatoid arthritis, *n* (%)	5 (5.88)	3 (5.36)	1.000
ANCA associated vasculitis, *n* (%)	1 (1.18)	3 (5.36)	0.345
**Comorbid conditions**			
Hypertension, *n* (%)	24 (28.24)	19 (33.93)	0.472
Diabetes, *n* (%)	8 (9.41)	5 (8.93)	0.923
Coronary heart disease, *n* (%)	0 (0.00)	6 (10.71)	0.008*

Tumor, *n* (%)	3 (3.53)	4 (7.14)	0.568

Supplementary Table S3 Multivariable logistic regression analysis of comorbidities in late-onset versus early-onset SLE patients.

**Condition**	**Predictor**	**aOR**	**95% CI for aOR**	***P* value**	**Notes (Per Manuscript Findings)**
Hypertension	Group	1.16	[0.47, 2.84]	0.752	The increased prevalence in late-onset SLE was significantly associated with increasing age itself (*P* = 0.009), not group status.
	Age,years	1.04	[1.01, 1.07]	0.009*	
Diabetes	Group	40.73	[3.02, 549.50]	0.005*	Late-onset SLE was independently associated with a significantly higher risk of diabetes (aOR = 40.73).
	Age,years	0.97	[0.90, 1.04]	0.387	
